# Persistent Iron Deficiency Anemia in Patients with Celiac Disease Despite a Gluten-Free Diet

**DOI:** 10.3390/nu12082176

**Published:** 2020-07-22

**Authors:** Gianpiero Stefanelli, Angelo Viscido, Salvatore Longo, Marco Magistroni, Giovanni Latella

**Affiliations:** Gastroenterology, Hepatology and Nutrition Division, Department of Life, Health and Environmental Sciences, University of L’Aquila, 67100 L’Aquila, Italy; giastefanelli@gmail.com (G.S.); angelo.viscido@univaq.it (A.V.); salvator.longo@gmail.com (S.L.); magistroni.marco@gmail.com (M.M.)

**Keywords:** celiac disease, iron deficiency anemia, anemia of chronic disease, gluten-free diet

## Abstract

Celiac disease (CD) is an autoimmune disorder characterized by intolerance to dietary gluten in genetically predisposed subjects. Iron deficiency anemia (IDA) is a common sign in CD, being the only abnormality in approximately 40% of celiac patients. A multifactorial etiology leads to IDA in CD. The two main causes are the villous atrophy of the mucosa at the site of iron absorption (the duodenum) and the resulting inflammation, which triggers the mechanism that leads to the anemia of chronic disease. Until now, it has been unclear why some patients with CD continue to have IDA despite a careful gluten-free diet (GFD) and the normalization of villous atrophy. Furthermore, some celiac patients are refractory to oral iron supplementation despite the healing of the mucosa, and they thus require periodic intravenous iron administration. The Marsh classification evaluates the degree of inflammation and villous atrophy, but it does not assess the possible persistence of ultrastructural and molecular alterations in enterocytes. The latter was found in CD in remission after adopting a GFD and could be responsible for the persistently reduced absorption of iron and IDA. Even in non-celiac gluten sensitivity, anemia is present in 18.5–22% of patients and appears to be related to ultrastructural and molecular alterations in intestinal microvilli. It is possible that a genetic component may also play a role in IDA. In this review, we evaluate and discuss the main mechanisms of IDA in CD and the possible causes of its persistence after adopting a GFD, as well as their therapeutic implications.

## 1. Introduction

Celiac disease (CD) is an autoimmune disease involving the small bowel mucosa that is triggered by the ingestion of gluten in genetically predisposed subjects [1,2]. The diagnosis of CD in adults is based on the evaluation of clinical manifestations, serology, genetics, and the histology of duodenal biopsies, which may reveal intraepithelial lymphocytosis (IEL), crypt hyperplasia, and moderate-to-severe villous atrophy [2,3]. Duodenal biopsies can be avoided in the pediatric population with positive serology, such as anti-tissue-transglutaminase antibodies (tTG) IgA > 10 times the upper limit of normal values and positive anti-endomysial antibodies (EMA). In these cases, HLA-DQ2/HLA-DQ8 assessment and symptoms are not obligatory for the CD diagnosis [2].

CD is a global health-care problem [2,4,5]. The prevalence of biopsy-confirmed CD is estimated at over 1% of the population in the Western world. It is noteworthy that the incidence of CD is continuously increasing all over the world. It is highest in females and children, although CD is also becoming a common diagnosis in adults [4,5,6,7,8].

The clinical presentation of CD has a wide spectrum of manifestations, both intestinal and extra-intestinal, involving one or more organs [2]. Several clinical categories of CD have been identified, including classic/typical CD (characterized by intestinal symptoms), atypical/subclinical CD (characterized by minor or extra-intestinal symptoms), and silent CD (characterized by no symptoms) [9]. The category of the potential or latent CD has also been established to indicate those patients with positive serology in the absence of any pathological change during the histologic evaluation of duodenal biopsies [10].

The clinical manifestations of CD can be directly related to autoimmunity or can indirectly derive from intestinal inflammation and/or malabsorption [11].

**Figure 1:**
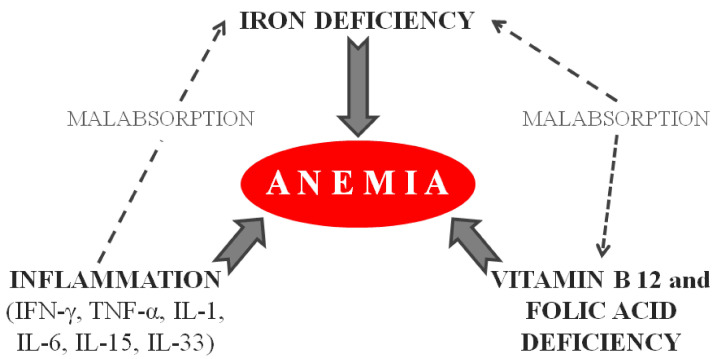
Main pathogenetic mechanisms of anemia associated with celiac disease.

Anemia is one of the most common clinical manifestations of CD and may be present in over half of patients at the time of diagnosis [12,13]. Anemia is usually hypo-regenerative (defined by a low reticulocyte count), reflecting the impaired absorption of iron and various vitamins such as folate and vitamin B_12_. Chronic inflammation may also play a role in the development of anemia [12,13] (Figure 1).

Iron deficiency anemia (IDA) is the most commonly recognized type of anemia in patients with CD [13,14]. IDA can be the only sign of CD [15], especially in patients with subclinical/atypical CD [9,10,16]. Iron is specifically absorbed in the duodenum, which is the site of major inflammation and injury in patients with CD. This explains why IDA is the major form of anemia in CD.

Folate and vitamin B_12_ malabsorption can also occur, but it mostly does in celiac patients with more extensive lesions [12,13,17]. The combination of iron and folate/vitamin B12 deficiency may cause specific morphological red cell changes, particularly dimorphic red cell populations.

Folate is primarily absorbed in the jejunum, which is commonly affected by CD. In patients with CD, the risk of folate deficiency is high, reaching up to 20–30% at CD diagnosis [13,18,19]. To be absorbed, the folate must be deconjugated by a brush border peptidase of enterocytes; the intestinal mucosal damage that occurs in CD can affect the activity of this enzyme, leading to folate deficiency.

The absorption of vitamin B_12_ requires the formation of a complex with the intrinsic factor produced by the stomach. The terminal ileum is the primary site of absorption of vitamin B_12_ [13].

At CD diagnosis, a prevalence of vitamin B_12_ deficiency of between 8 and 41% has been reported [19]. In CD, the causes of vitamin B_12_ deficiency are not yet fully clear. In addition to specific intestinal mucosal damage due to CD, they include other gastrointestinal disorders such as chronic autoimmune gastritis (with the reduced production of the intrinsic factor) frequently associated with CD, intestinal bacterial overgrowth (with the consumption of vitamin B_12_ by intestinal bacteria), and disorders of the distal tract of the small intestine (with the reduced absorption of vitamin B_12_).

In pediatric patients with CD, the range of the prevalence of low levels of folate and vitamin B_12_ has been found to be 15.7–18.3% and 4.3–8%, respectively [13,20,21].

Returning to the topic of IDA, blood loss from intestinal lesions can be considered an adjunctive cause that should be excluded.

It is worth noting that anemia in CD is caused not only by malabsorption or blood loss but also by other factors. Anemia of chronic disease (ACD), a form of anemia associated with chronic inflammation, may occur in up to a quarter of patients with CD [22,23,24]. Several inflammatory mediators, such as pro-inflammatory cytokines, can lead to ACD through three different mechanisms: iron reallocation outside the serum, defective erythropoietin production leading to the impaired proliferation and maturation of erythroid progenitors, and a reduction in the life span of erythrocytes [25] (Figure 1).

It has been reported that pro-inflammatory cytokines—in particular interferon-gamma (IFN-y), interleukin-6 (IL-6), and tumor necrosis factor-α (TNF-α)—trigger the synthesis of the iron regulatory hormone, hepcidin [26,27]. Hepcidin causes the degradation of ferroportin and inhibits the release of iron by macrophages and enterocytes [13,25,26].

**Figure 2:**
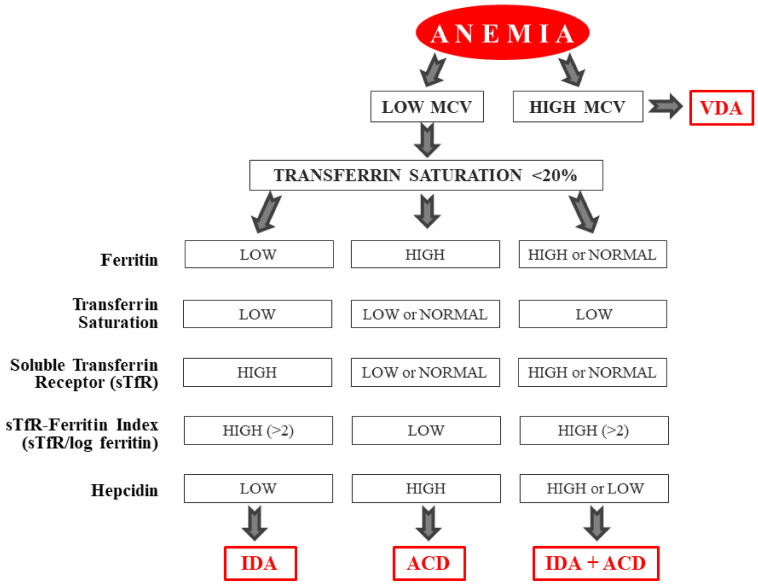
Characteristics of the different types of anemia associated with celiac disease (CD): iron deficiency anemia (IDA), anemia of chronic disease (ACD), and vitamin B_12_ and folic acid deficiency anemia (VDA).

It is likely that IDA and ACD can coexist in CD before diagnosis and before adopting a gluten-free diet (GFD), whereas after a prolonged period on a GFD, the role of ACD is likely to be minimal or absent. In clinical practice, it is possible to evaluate whether IDA or ACD prevails by using several laboratory parameters (Figure 2) [13,23,24].

It is noteworthy that IDA, like other clinical manifestations, can persist in a proportion of patients with CD that experience mucosal healing and the resolution of intestinal malabsorption after excluding gluten from the diet [13,14,28,29]. This review provides an overview of the pathogenic mechanisms of IDA that characterize CD, as well as a rationale for its management.

## 2. Materials and Methods

This review primarily focuses on published studies performed on IDA in patients with CD selected from a computer search of the literature (Medline/PubMed, Scopus, and Cochrane) from January 1990 to January 2020, performed by three authors (G.S., S.L., and M.M.), and using the following keywords: “celiac disease,” “anemia,” “microcytic anemia,” “macrocytic anemia,” “megaloblastic anemia,” “iron deficiency,” “micronutrient deficiencies,” “iron-deficient anemia,” ”IDA,” “anemia of chronic disease,” “ACD,” “persistent anemia in celiac disease,” “refractory anemia,” “gluten-free diet,” and “GFD.” Additional studies were identified through a manual review of the reference lists of the selected studies and review articles. Clinically significant full-text articles were selected for this review.

## 3. Results

### 3.1. Iron Deficiency Anemia and Relationship with Clinical and Histological Features

IDA is a relatively frequent condition caused by iron loss or reduced iron absorption. It is defined as reduced values of hemoglobin and mean corpuscular volume, as well as low serum iron and ferritin [30,31]. As ferritin increases in response to inflammation, a normal ferritin level does not exclude IDA in the presence of inflammation [30,31]. In the United States, IDA was reported in 2–5% of premenopausal women [32]. Rockey et al. studied, by colonoscopy and esophagogastroduodenoscopy, 100 adult patients with idiopathic IDA [33]. Out of these patients, 62 patients showed at least one lesion that may have caused blood loss, but over 30% of the patients had no signs of gastrointestinal bleeding [33]. IDA can be the result of an imbalance between iron loss and absorption, and it is often considered to be the result of an occult gastrointestinal bleeding that is detectable by a fecal occult blood test [34].

Since patients with IDA are more likely to have CD than the general population, serum screening tests for CD should always be performed in these patients. A cross-sectional study on 290 patients (age more than 12 years) diagnosed with IDA showed that tTGs were found in 32 (11%) of these patients (*p* = 0.0002) [35]. Shahriari et al. studied 184 children: 92 patients with IDA responsive to iron supplementation, 45 patients with IDA unresponsive to iron supplementation, and 47 controls [36]. They found that the frequency of positive antibodies for CD was higher in the second group compared with the other two groups (*p* < 0.001). They also showed that the frequency of potential CD was higher in patients unresponsive to iron supplementation than controls.

Data on the prevalence of IDA at the time of CD diagnosis and after starting a GFD, both in adult and pediatric patients with CD, are summarized in Table 1.

Kolho et al. reported IDA in 25% of Finnish adult patients at the time of CD diagnosis, all with partial or total villous atrophy [7]. Akbari et al. reported IDA in 52% of adult Iranian patients at the time of CD diagnosis, regardless of their histological Marsh grade [8]. In a cohort of 132 Italian adult patients with CD, IDA was found in 34% at the time of CD diagnosis, and anemia either improved or was completely corrected in most of these patients after one year on a GFD [22]. In a prospective observational study including 103 adult Indian patients with CD, IDA was found in 84 (81.5%) patients at the time of CD diagnosis [24].

A recent Turkish retrospective analysis of 195 adult patients at the time of CD diagnosis showed that anemia was found in 60.5% of patients (IDA in 53.3% of cases, folic acid deficiency in 38.4% of cases, vitamin B_12_ deficiency in 25.6% of cases, and ACD in 10.2% of cases) [37]. In an Italian multicenter study including 1026 patients with subclinical/silent CD, IDA was the most frequent extra-intestinal sign (39%) in this CD subtype, being found in 46% of adult CD patients and 35% of pediatric CD patients [38]. In a US study including 727 adult patients with CD, IDA was found in approximately 21% of patients [39]. In another retrospective study from the USA, the prevalence of IDA was assessed in both adult and pediatric patients with CD—IDA was markedly more frequent in adult celiac patients than in pediatric patients (48% versus 12%, respectively) [40]. An improvement in IDA was observed in both adult and pediatric patients after at least 24 months of a GFD (85% and 84%, respectively). In a recent study including 387 Italian children with CD, 134 (35%) patients had IDA at the time of CD diagnosis [41]. In a cohort of 505 adult patients with CD, 229 patients had IDA (45%); of these, approximately half were found to have persistent IDA after one year on a GFD and the histological normalization of the duodenal mucosa [42].

Duodenal villous atrophy impairs iron absorption, and IDA may be the only clinical sign in adults with CD [43]. Chronic gastritis, which is frequently present in patients with CD, can contribute to iron malabsorption [44]. Anemia was also found in potential CD, a condition without apparent duodenal damage. In a prospective study including 163 adult celiac patients, Saukkonen et al. compared patients with anemia (23%) versus patients without anemia (77%) at the time of CD diagnosis and then after one year on a GFD. They showed that CD was more severe in patients with anemia than in patients without anemia. At the time of CD diagnosis, the tTG median value was higher in those with than those without anemia (65 versus 26.4 U/mL, respectively; *p* = 0.007). Furthermore, at the time of CD diagnosis, the body mass index (BMI) was lower in the first than in the second group (22.1 versus 25.2 kg/m^2^, *p* = 0.002). After one year on a GFD, the villous height–crypt ratio was lower in patients with anemia than those without anemia (2.5 versus 1.9, respectively; *p* = 0.008) and IEL was higher in patients with anemia than without (33 versus 27 cells/100 epithelial cells, respectively; *p* = 0.054) [45]. In a cohort of 727 adult celiac patients, Abu Daya et al. showed that villous atrophy and bone mass density were worse in patients presenting with anemia than those presenting with diarrhea at CD diagnosis [39]. Similarly, Nurminen et al. compared three groups of celiac children (116 children with extra-intestinal manifestations at CD diagnosis versus 249 children with gastrointestinal symptoms at CD diagnosis versus 146 children diagnosed by a serum CD screening test that was performed for the presence of familiarity for CD) and demonstrated that the degree of villous atrophy was worse in CD patients with extra-intestinal manifestations at CD diagnosis. They reported that anemia was the second most frequent extra-intestinal manifestation (18%) after poor growth in celiac children, as well as that median hemoglobin values were slightly worse in the first group (12.3 g/dL) than in the second (12.4 g/dL) and third groups (12.6 g/dL) (*p* = 0.032) [46]. Considering that the absence of typical gastrointestinal symptoms can delay the diagnosis of CD, it is probable that this delay is the cause of their worse villous atrophy at CD diagnosis, as well as of the more severe anemia. In a retrospective study including 109 young celiac patients (age ≤ 20 years old), Bhadada et al. showed that anemia was more frequent in patients diagnosed with CD alone than in patients diagnosed with CD and type 1 diabetes mellitus: 80.9% versus 45% (*p* < 0.01), respectively. They reported that the lag time between the onset of symptoms and the diagnosis of CD was higher in patients with CD alone (48.8 versus 20.2 months, respectively; *p* < 0.05) [47]. In a case series of 103 adult celiac patients, Berry et al. found anemia in 93% of patients at CD diagnosis, IDA in 81.5%, vitamin B_12_ deficiency in 13.6%, folate deficiency in 10.7%, mixed nutritional deficiency in 16.5%, and ACD in 3.9% [24]. Hemoglobin and ferritin values were lower in patients with severe villous atrophy than in those with mild villous atrophy (mean hemoglobin values were 8.2 versus 9.6 g/dL, *p* = 0.004; median ferritin values were 5 versus 20 ng/mL, *p* = 0.002, respectively). The hypothesis that the degree of villous atrophy correlates with anemia severity was partially confirmed by Harper et al. After analyzing a cohort of 405 adult celiac patients at the time of CD diagnosis, they showed that degree of villous atrophy did not influence the proportion of anemic patients. Conversely, there was a significant difference in the proportion of patients with iron deficiency (34% of patients with subtotal/total villous atrophy versus 13% of patients with partial villous atrophy: *p* > 0.001) [48]. Annibale et al. studied 26 adult celiac patients by duodenal biopsies and hematological tests [49]. They demonstrated a significant inverse correlation (r = −0.7141 and *p* = 0.0003) between an increase in hemoglobin concentration and a decrease of histological scores of duodenal lesions after adopting a GFD. They concluded that the hemoglobin value normalizes 6–12 months after starting a GFD alone as a consequence of the normalization of alterations in the intestinal mucosa. Conversely, after 12 months on a GFD, only 50% of patients had recovered from iron deficiency [49]. In a study including 102 children with CD, Repo et al. showed that median values for hemoglobin, iron, ferritin, and transferrin saturation were significantly lower in the case of total villous atrophy compared with partial/subtotal villous atrophy, potential CD, and controls [50]. Anemia was found in 63% of patients with total villous atrophy, in 22% of patients with partial/subtotal villous atrophy, in 15% of patients with potential CD, and in 0% of controls.

Studies aimed at understanding why there is a partial response to a GFD are lacking. Efthymakis et al. investigated the diagnostic role of small bowel capsule endoscopy in 26 adult celiac patients with persistent IDA, despite them following a GFD [51]. In 23% of these celiac patients, capsule endoscopy showed significant findings (such as refractory CD, Crohn’s disease, angiodysplasia, and lymphangiectasia) that could explain IDA persistence. In a randomized controlled trial including 34 children or adolescent celiac patients, Feruś et al. analyzed the influence of prebiotics on iron homeostasis [52]. At the time of the enrollment, patients had been on a GFD for at least six months and were not anemic. Patients were randomized into two groups of receiving prebiotic (oligofructose-enriched inulin: Synergy 1) at 10 g/day or a placebo for 12 weeks. The serum hepcidin concentration after the intervention decreased significantly by 60.9% (*p* = 0.046) in the Synergy 1 group, whereas no significant difference was observed in the placebo group. The reduction of hepcidin was probably due to the potential anti-inflammatory effect of prebiotics. However, no differences in morphological or biochemical blood parameters (including ferritin, hemoglobin, and C-reactive protein) were observed after intervention in either group [52].

### 3.2. Underlying Causes of IDA in Celiac Disease

The leading causes of IDA in celiac patients include iron malabsorption, chronic intestinal inflammation that is typical of CD, and the concomitant presence of other inflammatory bowel diseases (e.g., inflammatory bowel diseases (IBD) and giardiasis) or extraintestinal diseases (e.g., arthritis and dermatitis), as well as gastrointestinal occult blood losses (e.g., concomitant intestinal parasites, drug-induced enteropathies, intestinal neoplasia, and gastric infection with *Helicobacter pylori*) and increased need for iron (e.g., in pregnancy and in competitive sports). A non-negligible role in causing IDA in CD patients could be played by an inadequate low-iron diet, given that a GFD does not generally include enriched/fortified iron from wheat-based foods.

#### 3.2.1. Iron Malabsorption

Iron is an essential micronutrient for all cells because it is required for erythropoiesis, oxidative metabolism, and enzymatic activities, and it is a cofactor for mitochondrial respiratory chain enzymes, the citric acid cycle, and DNA synthesis. It also promotes the growth of immune system cells [13].

**Figure 3:**
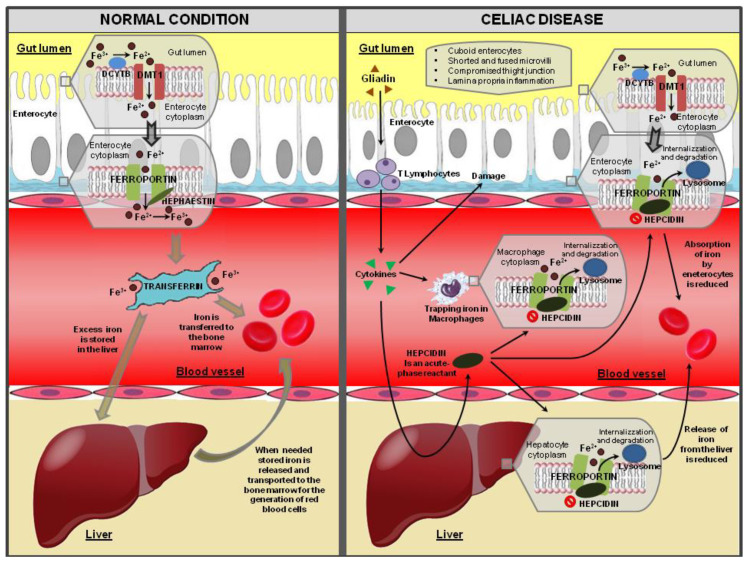
Mechanisms of iron deficiency anemia in celiac disease.In normal conditions, iron is absorbed in the gastrointestinal tract and then delivered to transferrin for transport to developing red cells; excess iron is stored in hepatocytes. In celiac disease, due to the atrophy of the intestinal mucosa, there is a reduced intestinal absorption of iron and therefore reduced iron delivery to developing red cells. Furthermore, the concomitant inflammation of the mucosa induced by gluten causes the increased release of cytokines, which can induce increased hepatic hepcidin production; hepcidin inhibits ferroportin, the main iron-export protein, and it consequently reduces iron release from enterocytes, hepatocytes, and circulating macrophages. DMT1: divalent metal transporter 1; DCYTB: duodenal cytochrome B. Adapted from DeLoughery et al. [55].

Non-heme iron is mainly present in the diet as the ferric form (Fe^3+^). The iron to be absorbed must be converted from the Fe^3+^ form to the ferrous form (Fe^2+^) by the combined action of duodenal cytochrome B (DCYTB), a ferrireductase that is present on the apical membrane of duodenal enterocytes, or dietary reducing agents such as ascorbic acid [13,53,54,55]. Iron in the Fe^2+^ form is then transported across the apical duodenal membrane by the iron symporter, divalent metal transporter 1 (DMT1) [13,53,55]. DMT1 is a protein found on the brush border membrane and can carry iron and many other divalent metals [54]. Thus, it is stored inside enterocytes [13,14,55]. Fe^2+^ is subsequently transferred to the blood via the iron exporter, ferroportin, which is also called metal transporter 1 (MTP 1); MTP 1 is localized in the basolateral membrane (Figure 3). For transport in the bloodstream, iron is bound to transferrin, which can transport two ferric (Fe^3+^) ions to distant target tissues. Prior to transferrin binding, ferrous (Fe^2+^) ions must be reconverted to ferric (Fe^3+^) ions. Excess iron can be deposited in the liver, and then it can be released and used later [14,55]. Iron homeostasis is regulated by hepcidin, which is a hormone synthesized by hepatocytes and secreted into the blood [56,57]. By internalizing and degrading ferroportin, hepcidin reduces the iron inflow into the blood plasma from the duodenum, from macrophages involved in recycling senescent erythrocytes, and from hepatocytes.

Iron absorption is reduced in patients with CD due to duodenal villous atrophy (the duodenum is the specific site of iron absorption) [58]. Villous atrophy is caused by gliadin or its derived peptides, such as p31–49 and p57–73 [26,59]. p31–49, through IL-15, induces the expression of a stress molecule, major histocompatibility complex class I-related chain A (MICA), on the surface of enterocytes. MICA activates natural killer cell receptors (NKG2D) on IEL: This interaction causes IEL cytotoxicity, leading to villous atrophy [59]. Furthermore, gluten peptide p57–73 is presented to mesenteric lymph node T cells by HLA-DQ2 on antigen-presenting dendritic cells. Thus, antigen-specific mesenteric T cells come back to the bowel by engaging specific cell adhesion molecules, inducing the release of cytokine (mainly IFN-γ), ultimately resulting in cell death [26]. Thus, epithelial cytotoxicity and the subsequent villous atrophy is the result of at least two mechanisms: indirectly through cytokines (especially IFN-γ) released by specific T cells and directly by IEL via MICA/NKG2D interactions [26]. On one hand, the abnormal immune activation induced by gluten peptides and consequent epithelial damage affects the absorption of iron; on the other hand, it induces the chronic inflammation of the mucosa, which in turn affects the metabolism of iron (Figure 3) [55].

#### 3.2.2. Chronic Inflammation

Inflammatory states are often accompanied by microcytic anemia, and they can lead to the alteration of iron homeostasis [60]. At least two mechanisms lead to anemia in the case of inflammation [25,55,60]. First, inflammatory cytokines suppress erythropoietin production from the kidneys. Second, the availability of iron is reduced due to the hepcidin, which is an acute-phase reactant [55]. Hepcidin, which binds and down-regulates ferroportin, prevents the transfer of iron from enterocytes and from body stores to blood for the generation of red blood cells [55,61] (Figure 3).

Increased serum levels of acute-phase proteins, such as the C-reactive protein (CRP), are rare in CD, although the gliadin-dependent activation of mononuclear cells of the lamina propria mucosa causes the hyperproduction of pro-inflammatory cytokines, such as IFN-γ and IL-6 [62,63,64], which are both mediators of anemia and systemic inflammation [65,66]. In CD, IFN-γ is the main cytokine secreted by inflammatory cells in the intestinal mucosa after exposure to gluten [62,63,64]. IFN-γ, TNF-α, IL-1, IL-6, IL-15, and IL-33 are increased in CD and are considered signs of active disease [22]. IL-6 and IFN-γ inhibit the mRNA expression of the transferrin receptor, and IFN-γ favors iron retention within monocytes and suppresses erythropoiesis via IL-15 [65,66,67]. IL-15 is implicated in the pathophysiology of the inflammation in CD and is partly responsible for prolonged inflammation [68]. These cytokines are released into the circulation and act on the liver, causing the induction of hepcidin, whose role is to inhibit the duodenal absorption of dietary iron (Figure 3). These cytokines also up-regulate the DMT-1 present on macrophages, thus increasing their iron absorption, but they simultaneously down-regulate the ferroportin (which is a transmembrane exporter of iron). These mechanisms prevent the release of iron from the macrophages into the circulation, and the net effect is the entrapment of the iron in the reticuloendothelial system [25]. This finding suggests that gluten-induced inflammatory responses in the intestinal mucosa may have a primary role in determining the malabsorption of iron, the dysregulation of iron homeostasis, and ineffective erythropoietin production, thus leading to anemia in some patients (Figure 1 and Figure 3). The improvement of the inflammation and atrophy of the intestinal mucosa that can be obtained with GFD can lead to a progressive correction of anemia, both by improving iron absorption and by reducing the effects of various inflammatory mediators on iron homeostasis and erythropoiesis.

#### 3.2.3. Other Causes of Iron Deficiency Anemia in Celiac Patients

Causes of iron loss must be excluded, even in celiac patients. Causes of bleeding from the lower digestive tract should be excluded by an occult blood stool test and subsequent colonoscopy [69]. Esophagogastroduodenoscopy must be performed to evaluate erosive gastritis, *H. pylori* infection, esophagitis, erosive duodenitis, and microscopic enteropathy. The association between IDA in celiac patients and *H. pylori* infection remains controversial [44,70,71,72,73,74,75].

Cuoco et al. assessed the relationship between *H. pylori* and IDA in 362 adult patients with CD, and they found *H. pylori* infection in 21% of cases [72]. In a cohort of 213 adult patients with CD, Capannolo et al. found *H. pylori* infection in 32.1% of cases [44]. Demir et al. reported that 42% of pediatric patients with CD were infected with *H. pylori*, among which 47% had IDA [73]. On the other hand, Simondi et al. showed that the frequency of *H. pylori* infection did not significantly differ between celiac adult patients with or without IDA [74]. Given the strong association between IDA and *H. pylori*, taking gastric biopsies to diagnose *H. pylori* infection should be a routine part of the diagnostic workup to identify the causes of IDA in patients with CD [75,76].

An active *H. pylori* infection is found in over 50% of patients with unsolved IDA [69,77,78]. A meta-analysis by Muhsen et al. showed an increased risk for IDA in patients (both adults and children) with active *H. pylori* infection [79]. In their meta-analysis including 16 studies with a total of 956 patients (both adults and children), Yuan et al. concluded that *H. pylori* eradication could resolve IDA [80]. The pathogenesis of IDA related to *H. pylori* is not fully known [79], and various mechanism have been proposed: chronic gastrointestinal bleeding due to gastric mucosa erosions, the use of iron by bacteria for growth and proliferation, a reduced concentration of ascorbic acid in the gastric juices, and the upregulation of proinflammatory cytokines and hepcidin (a key regulator of iron homeostasis) [69,77,79,81,82,83,84]. In vitro studies have shown that iron-repressible outer membrane proteins are expressed by *H. pylori* and could be involved in heme binding and/or uptake [85]. Kirschener and Blaser proposed a mathematical model to explain this form of chronic parasitism: *H. pylori* leads to an inflammatory state that damages tissue, resulting in nutrient release, including iron. In this model of a parasitic relationship, the bacterium competes with the host for iron, progressively decreasing the iron stores, with IDA ensuing over time [86].

Microscopic enteropathy with an increased number of intestinal IELs can be associated with IDA. The increase in IELs, in addition to being a characteristic histological feature of CD, has been reported in various other disorders such as *H. pylori* infection, *Giardia* infection, IgA deficiency, hypogammaglobulinemia, autoimmune enteropathy, common variable immune deficiency, eosinophilic gastroenteritis, collagenous sprue, and Crohn’s disease [87,88].

Potential infection with *Giardia lamblia* must be considered as a possible cause of IDA; although giardiasis is rare, its histopathological and serological picture may resemble that of CD. In some cases, *G. lamblia* can survive for long periods and can lead to partial or complete villous atrophy and crypt hyperplasia, which closely resemble CD [89,90]. It has also been shown, albeit only in case reports, that IgA antigliadin antibodies and IgA tTG can be exclusively positive because of the presence of *G. lamblia* [91]. Giardiasis is the most common human protozoan infection with a fairly high seroprevalence even in industrialized countries; its symptoms resemble those of CD, and it could also manifest itself as IDA [92].

Other rare causes of anemia that can be associated with CD include hemolysis, myelodysplastic syndrome, chronic renal insufficiency, aplasia (mainly drug-induced), congenital hemoglobinopathy, and erythropoiesis disorders.

### 3.3. Persistence of Anemia in Patients with CD Despite Adopting a GFD

The factors that could explain the persistence of anemia, and in particular of IDA, in patients with CD despite following a GFD have not yet been identified or clarified. Excluding non-adherence to a GFD as the main cause, the presence of ultrastructural and/or molecular alterations of enterocytes or specific genetic factors would seem to be involved in the persistence of IDA after adopting a GFD and oral iron supplementation.

#### 3.3.1. Ultrastructural and Molecular Alterations of Enterocytes

Concerning anemia that is refractory to oral iron supplementation in celiac patients despite the recovery of duodenal mucosa after adopting a GFD, it is conceivable that the ultrastructural and molecular alteration of the enterocytes could play a role. In many patients with CD on a GFD, the histologic evaluation of duodenal biopsies finds no evidence of significant mucosal lesions. However, in some adult patients mucosal healing is not always complete. In a study on 27 pediatric celiac patients (and five controls), Stenling et al. investigated duodenal biopsies by low, medium, and high power scanning electron microscopy, both before and after adopting a GFD. They demonstrated the persistence of ultrastructural lesions in enterocytes, such as disrupted and decreased glycocalyx, and irregular or absent microvilli after adopting a GFD when biopsies were investigated by medium or high power scanning electron microscopy, whereas low power scanning electron microscopy did not detect these lesions. Of the 27 patients (13 girls and 14 boys), seven were between 6 and 30 months, six were between three and seven years, and four were over 10 years at the time of the first biopsy. Dietary instructions were given to the patients and their families by a trained dietician [93]. Similarly, in a study including 70 children (34 at CD diagnosis, 28 celiac patients after two-to-seven years of a GFD, and eight controls), Dyduch et al. used transmission electron microscopy to investigate various ultrastructural features, such as the thickness of the glycocalyx and the number and appearance of the microvilli. They observed that the glycocalyx was thin or completely absent and that the height of the microvilli remained reduced in some celiac patients after adopting a GFD [94]. In an electron microscopy study of 13 celiac patients, Magliocca et al. found ultrastructural alterations of enterocytes that were not detected using low-resolution light microscopic techniques [95]. Overall, the results of the above studies suggest that the persistence of ultrastructural alterations in the enterocytes even after a prolonged period on a GFD may be responsible for IDA that is refractory to oral iron supplementation in CD patients on a GFD and with histological findings of the complete regression of the atrophy of the duodenal mucosa.

Ultrastructural and molecular alterations of enterocytes would also appear to be responsible for IDA in patients with non-coeliac gluten sensitivity (NCGS). IDA was found in approximately 20% of patients with NCGS [96,97]. NCGS is a disorder characterized by the appearance of reported gluten-related symptoms that disappear after adopting a GFD. A gluten challenge test is necessary to confirm NCGS, and CD and wheat allergy must first be ruled out [96]. The exact pathogenetic mechanisms of NCGS remain unclear. Alterations in intestinal permeability leading to the excessive absorption of gluten-derived peptides and an abnormal wheat-induced innate reaction are possible mechanisms. The prevalence of NCGS is not yet known, and it is probably higher than that of CD [98]. In a cohort of 392 consecutive patients complaining of gluten-related symptoms, Capannolo et al. reported that the prevalence of NCGS was 6.88% (27 patients). Out of 27 patients, five (18.5%) showed IDA [96]. In a multicenter (38 Italian centers) survey of 486 patients with NCGS, Volta et al. found anemia (due to both iron and folic acid deficiency) in 22% of patients [97]. Given the absence of the typical histological alterations of CD in duodenal biopsies obtained from patients diagnosed with NCGS, anemia in NCGS could be related to the ultrastructural and molecular alteration of enterocytes. Sbarbati et al. evaluated intestinal specimens from 14 healthy controls and seven patients with gluten sensitivity and normal histology using both scanning and transmission electron microscopy. They revealed alterations of the enterocyte brush border with a significant reduction of the height of microvilli in four out of seven patients with gluten sensitivity—alterations that were not detected by conventional light microscopy [99]. Meanwhile, Barbaro et al. investigated the zonulin serum levels in 185 patients (86 with NCGS, 59 with diarrhea-predominant irritable bowel syndrome, 15 with CD, and 25 controls). They showed that zonulin serum levels were significantly increased both in CD and in NCGS compared with healthy controls—these zonulin levels decreased only partially after adopting a GFD [100].

#### 3.3.2. Genetic factors

Several studies have evaluated the possible genetic predisposition to IDA in CD. Barisani et al. evaluated iron regulatory proteins in 25 celiac patients compared to 10 controls and six iron-deficient patients. They collected duodenal biopsies from all these patients and investigated DMT1, DCYTB, ferroportin 1 (FP1), hephaestin, and transferrin receptor 1 (TfR1) expression, as well as iron regulatory protein (IRP) activity. They showed that in all patients, including celiac patients, body iron stores mainly influenced the expression of these proteins. They demonstrated that DMT1, FP1, hephaestin, and TfR1 expression are similarly regulated in CD and non-CD patients. Thus, CD alone did not influence the expression of these proteins involved in iron absorption [101]. Similarly, Sharma et al. obtained duodenal biopsies from untreated celiac patients with or without IDA and from anemic and non-anemic patients with normal duodenum. The aim of the study was to investigate the expression of a protein involved in iron transport [102]. Consistent with Barisani et al. [101], they showed that DMT1 and ferroportin expression was increased in patients with IDA, regardless of the presence of CD. Furthermore, they found that ferritin expression was only increased in celiac patients with IDA, whereas it was normal in non-celiac patients. Ferritin overexpression is likely to be due to mucosal inflammation; activated IELs produce TNF-α, which blocks iron export from cells [102]. Tolone et al. investigated the role of DMT1 polymorphisms in 387 celiac children, comparing them to 164 controls. In particular, they studied the role of the DMT1 IVS4 + 44C > A variant. They showed that DMT1 expression was higher in CC homozygous patients than in AA homozygous and CA heterozygous patients, regardless of villous atrophy. Thus, the A-allele was more frequent in celiac patients with IDA than without IDA. As a result, if the DMT1 IVS4 + 44-AA genotype is present, the risk of developing anemia increases four-fold, regardless of the degree of atrophy. In fact, the A-allele limits the overexpression of DMT1 that is required in the case of iron deficiency [41]. Barisani et al. [103]; subsequently, De Falco et al. [42] investigated the role of hemochromatosis (HFE) gene expression and mutations in iron metabolism in CD. They hypothesized that HFE mutations, which increase bowel iron absorption, could protect against IDA in CD. Barisani et al. showed that in 203 Italian patients with untreated CD, HFE mutations did not protect against IDA, whereas iron deficiency seemed to be related to the severity of duodenal lesions [103]. De Falco et al. also studied the possible influence of HFE C282Y and H63D and TMPRSS6 A736V variants on the pathogenesis of IDA in 505 celiac patients at the time of diagnosis and after one year on a GFD, compared to 539 control subjects [42]. In contrast to Barisani et al. [103], they showed that HFE mutations protect against IDA in celiac patients. Furthermore, they confirmed that the oral iron response depends on TMPRSS6, which modulates hepcidin action. In fact, at follow-up, the allele frequency of the A736V mutation was higher in celiac patients with persistent IDA than in non-anemic celiac patients. Thus, the TMPRSS6 genotype could provide information about the response to oral iron therapy and a GFD in anemic celiac patients [42]. On the other hand, Elli et al. found a significantly higher percentage of TMPRSS6 mutations in celiac patients compared to controls, while no differences were found between IDA and non-IDA in CD patients [104]. Further studies will be needed to establish the real role of specific genetic factors in determining the persistence of IDA in patients with CD despite adopting a GFD.

## 4. Conclusions

A multifactorial etiology may lead to anemia in celiac patients [13,49]. At the time of celiac disease diagnosis, the two main causes of anemia are the villous atrophy of the mucosa in the proximal small intestine (which leads to impaired absorption of various nutrients—especially iron) and the mucosal inflammation that triggers the mechanism that leads to ACD. IDA in CD may also be a consequence of the reduced expression of different proteins that regulate iron absorption.

In patients with CD and on a gluten-free diet, the persistence of IDA can be due to various conditions, such as a lack of adherence to the GFD, the involuntary intake by the patient of food erroneously contaminated with gluten, to the intake of a low-iron GFD (a GFD does not generally include enriched/fortified iron from wheat-based foods), or to the persistence of atrophy of the intestinal mucosa, which leads to an impairment of iron absorption. In patients with CD and persistent IDA despite a careful GFD, the persistence of villous atrophy should first be excluded by the histology of duodenal biopsies. If mucosal healing has occurred, possible occult blood loss from other parts of the intestinal tract should be investigated by colonoscopy and then bowel capsule endoscopy to exclude other intestinal diseases. In addition, the physiological causes of IDA, such as iron loss through copious menses in fertile women, gastrointestinal occult blood loss, and urinary iron losses due to intense exercise-induced hemolysis in athletes, should be considered [55]. Furthermore, some patients are refractory to oral iron supplementation and require periodic intravenous iron administration [105]. Several conditions can cause refractoriness to oral iron supplementation. First, one should consider the possible side effects of supplementation, such as abdominal discomfort, stomach pain, constipation, and diarrhea, which can lead to reduced iron intake. In this regard, it has been reported that some iron formulations such as sucrosomial iron (a ferric pyrophosphate covered by a phospholipid and sucrester membrane) and feralgine (a solution of ferrous bisglycinate chelate and sodium alginate) can be effective in providing iron supplementation in a celiac patient intolerant to iron sulfate, allowing for good intestinal absorption independently of the DMT-1 carrier [13,106,107]. Tea, coffee, calcium, and fiber can also reduce intestinal iron absorption. Conversely, taking vitamin C increases iron absorption, and the intake of iron with meat proteins can also increase the intestinal absorption of iron. Of note, the rate of absorption of iron from heme sources is markedly higher (>10 times) than that of iron from non-heme sources. It is worth noting that enterocyte iron absorption is saturable and that one high iron dose reduces the absorption of subsequent doses. In patients with persistent IDA despite duodenal mucosa recovery and the administration of oral iron supplementation, ultrastructural alterations of the enterocytes that can impair iron absorption should also be considered. However, other studies are needed to clarify the persistence of IDA in CD despite a GFD. Meanwhile, the administration of intravenous iron is necessary for these patients. Furthermore, the treatment of any concomitant extra-intestinal inflammatory diseases can contribute to the reduction of the negative effects of the related inflammatory mediators on iron homeostasis and erythropoiesis [55]. It is also likely that a genetic component is important, and in the near future, we will move closer and closer to personalized therapy. Variants of some genotypes may induce a poor response to dietary and supplemental iron therapy and may predict the persistence of IDA despite iron treatment and GFD. One of the therapeutic goals in CD is the improvement of the health-related quality of life and the resolution of the sensation of chronic fatigue that is correlated with the severity of anemia. As most celiac patients are young, these patients may have higher physical and cognitive demands; therefore, the normalization of iron and hemoglobin levels should be sought. In particular, it is known that in children, both the developing brain and cognitive functions, are sensitive to iron deficiency, regardless of whether anemia is present or not.

## Figures and Tables

**Table 1:** Prevalence of IDA at CD diagnosis and after a gluten-free diet (GFD), both in adult and pediatric patients.

**Adults Patients**							
**Study (Year)**	**Study Location**	**Study Design**	**Number of CD Patients**	**Men N.(%)**	**Follow-up**	**CD patients with IDA before the start of a GFD *N*. (%)**	**CD patients with IDA after the start of a GFD (%)**
Kolho et al. (1998) [7]	Finland	Cross-sectional	8	1 (13)	n.a.	2 (25)	n.a.
Akbari et al. (2006) [8]	Iran	Cross-sectional	27	14 (52)	n.a.	14 (52)	n.a.
Bergamaschi et al. (2008) [22]	Italy	Prospective Cohort	132	47 (36)	12 months	45 (34)	n.a.
Berry et al. (2018) ** [24]	India	Prospective cohort	103	48 (47)	n.a.	84 (82)	n.a.
Binicier et al. (2020) [37]	Turkey	Retrospective Cohort	195	44 (23)	58 months *	104 (53)	n.a.
Bottaro et al. (1999) [38]	Italy	Cross-sectional	313	n.a.	n.a.	145 (46)	n.a.
Abu Daya et al. (2013) [39]	USA	Cross-sectional	727	203 (28)	n.a.	152 (21)	n.a.
Sansotta et al. (2018) [40]	USA	Retrospective Cohort	327	n.a.	24 months *	157 (48)	(15)
De Falco et al. (2018) [42]	Italy	Prospective Cohort	505	106(21)	12 months	229 (45)	(21)
**Pediatric Patients**							
**Study (Year)**	**Study Location**	**Study Design**	**Number of CD Patients**	**Men N.(%)**	**Follow-up**	**CD patients with IDA before the start of a GFD *N*. (%)**	**CD patients with IDA after the start of a GFD (%)**
Bottaro et al. (1999) [38]	Italy	Cross-sectional	485	n.a.	n.a.	169 (35)	n.a.
Sansotta et al. (2018) [40]	USA	Retrospective Cohort	227	n.a.	31 months *	27 (12)	(16)
Tolone et al. (2017) [41]	Italy	Cross-sectional	387	152 (39)	84 months	134 (35)	n.a.

Note: N., number; CD, celiac disease; GFD, gluten-free diet; IDA, iron deficiency anemia; n.a., data not available; * mean; and ** age > 12years.

## Abbreviations

CD	Celiac disease
IDA	Iron deficiency anemia
GFD	Gluten-free diet
IEL	Intraepithelial lymphocytosis
tTG	Anti-tissue-transglutaminase antibodies
EMA	Anti-endomysial antibodies
ACD	Anemia of chronic disease
IFN-y	Interferon-gamma
TNF-α	Tumor necrosis factor-α
IBD	Inflammatory bowel diseases
Fe^3+^	Ferric form
Fe^2+^	Ferrous form
DCYTB	Duodenal cytochrome B
DMT1	Divalent metal transporter 1
MTP 1	Metal transporter 1
MICA	Major histocompatibility complex class I-related chain A
NKG2D	Natural killer cell receptors
CRP	C-reactive protein
H. pylori	Helicobacter pylori
IELs	Intestinal intraepithelial lymphocytes
NCGS	Non-coeliac gluten sensitivity
FP1	Ferroportin 1
TfR1	Transferrin receptor 1
IRP	Iron regulatory protein
HFE	Hemochromatosis
